# Effect of Carbonation on Abrasion Resistance of Alkali-Activated Slag with Various Activators

**DOI:** 10.3390/ma12172812

**Published:** 2019-09-02

**Authors:** Hyeong-Ki Kim, Keum-Il Song, Jin-Kyu Song, Jeong Gook Jang

**Affiliations:** 1 School of Architecture, Chosun University, 309 Pilmun-daero, Dong-gu, Gwangju 61452, Korea; 2 School of Architecture, Chonnam National University, 77 Yongbong-ro, Buk-gu, Gwangju 61186, Korea; 3 Division of Architecture and Urban Design, Institute of Urban Science, Incheon National University, 119 Academy-ro, Yeonsu-gu, Incheon 22012, Korea

**Keywords:** abrasion resistance, carbonation, alkali-activated slag, activators, strength

## Abstract

The effect of carbonation on the abrasion resistance of alkali-activated slag (AAS) was investigated. Various activator sets were selected for synthesizing AAS specimens, and the compressive strength was measured before and after carbonation. The abrasion resistance of the specimens was measured in accordance with the ASTM C944 test method. The relationship between the mass loss caused by abrasion and compressive strength was analyzed to understand the effect of matrix strength on abrasion resistance. Test results showed that the decrease in compressive strength of AAS specimens by carbonation reduced their abrasion resistance. In addition, the abrasion resistance of AAS before and after carbonation was sensitively influenced by activator type. It can be concluded that additional caution is required when using AAS where abrasion may have occurred.

## 1. Introduction

Alkali-activated slag (AAS), an alternative binder material, has various advantages over ordinary Portland cement (OPC). Previous studies reported that AAS binder has excellent resistance against acid corrosion and sulfate expansion compared to conventional OPC [1,2,3,4,5]. This is attributed to the very dense microstructure of the AAS and its ability of ion binding [4,5]. Therefore, it is expected that AAS can be used to manufacture cementitious products for sewage pipes or sealing plaster, which are exposed to environments rich in acid and sulfate. However, these products also face other types of degradation phenomena, such as abrasion and/or carbonation. For example, sewage pipes are subjected to wear due to the various solids suspended in running water, in addition to being affected by acids and sulfates [6]. There is an only limited study on the abrasion resistance of AAS, and it has been confirmed that the abrasion resistance of AAS can vary significantly depending on the type of activator and curing regime [7,8]. However, there is no previous study on the effect of carbonation on the abrasion resistance of AAS. AAS has been known to have poor resistance against carbonation, which can cause shrinkage and degradation of mechanical strength [9,10]. From the previous findings, it is expected that if AAS matrices were carbonated, their abrasion resistance might be reduced consequently. In the present study, therefore, the effect of carbonation on the abrasion resistance of AAS was evaluated. Various types of activator were selected to synthesize AAS samples, and compressive strength of non-carbonated and carbonated samples were compared. The abrasion resistance of specimens with and without carbonation was measured by the ASTM C944 test method. The relationship between the compressive strength and mass loss caused by abrasion was analyzed to understand the abrasion resistance of AAS materials.

## 2. Experimental Procedure 

### 2.1. Materials and Mix Proportions

Blast furnace slag with the specific gravity and Blaine fineness of 2.90 and 4235 cm^2^/g, respectively, satisfying the ASTM C989 Grade 100, was used as a precursor. In this study, various “basic activator sets” and “additional binders” were selected. The “basic activator set” represents combinations of several activators resulting in the best strength and proper setting time, as confirmed by the previous study [11]. The three types of basic activator sets are named as follows: “A1” is a blend of sodium hydroxide (NaOH) and sodium carbonate (Na_2_CO_3_) in the ratio of 4:3 by mass; “A2” is sole sodium metasilicate (Na_2_SiO_3_·5.5H_2_O); and “A3” is a combination of calcium hydroxide (Ca(OH)_2_), sodium sulfate (Na_2_SO_4_), and sodium (hexa-) fluorosilicate (Na_2_SiF_6_) in a mass ratio of 7:3:2. The reason for preparing various mix proportions of AAS was to understand the general difference of the abrasion resistance of AAS with that of OPC after carbonation, rather than to compare the performance of each basic activator sets. The “additional binder” represents the materials expected to improve the resistance of AAS to carbonation. For this purpose, two types of material, a circulating fluidized bed combustion (CFBC) bottom ash and magnesium hydroxide (Mg(OH)_2_), were selected. The portlandite (Ca(OH)_2_) and free lime (f-CaO) in the CFBC ash are expected to bind carbonate ions as well as activate slag [12,13]. In addition, it was reported that Mg(OH)_2_ could play a similar role to Ca(OH)_2_ [14].

The slag and CFBC ash were supplied by steel works of the POSCO C&C, Inc. (Gwyangyang, South Korea) and the Gunsan power plant in South Korea, respectively. A sieve analysis result showed that approximately 99% of the CFBC bottom ash passed through a 1.25-mm sieve and 7% passed through a 105-μm sieve. The specific gravity of CFBC ash, measured by the ethanol method, is 2.72. All chemical products were obtained from the Honam Chemical, Inc. (Seoul, South Korea), and their purities were higher than 96%. For comparison of performance, a Type I OPC satisfying ASTM C150-17 standard was used. The results of the X-ray fluorescence (XRF) analysis of the slag, OPC, and CFBC ash are listed in Table 1. The X-ray diffraction (XRD) spectra and particle size distributions of the slag, OPC and CFBC ash, which measured by laser diffraction method in accordance with ISO 13320-1, are reported in previous studies conducted by authors [15,16]. Note that main crystalline structures detected by XRD spectra in CFBC ash are quartz, anhydrite, free lime and portlandite. River sand with a specific gravity of 2.65, water absorption ratio of 0.27 and fineness modulus of 2.75 was used as a fine aggregate.

Mix proportions of samples tested in this study are listed in Table 2. Note that the AAS mixtures in this study were designed as a “one-part” or “just-add-water” type [17]. In one-part mixtures, only dry materials containing slag and powder type activators were pre-mixed prior to the addition of water, similar to the preparation of OPC mixture. This type of mixture has greater potential than the conventional two-part type, especially in cast-in-situ applications. The water-to-binder ratio (w/b) and sand-to-binder ratio for all mixtures (OPC and AAS) were fixed to 0.485 and 2.45, respectively. Note that the w/b of 0.485 was determined in accordance with the ASTM C109 standard. It was considered the slag blended with “primary activators” as “binder set” and some portion of this binder set was replaced with additional binder by weight. Four levels of contents of the additional binders, i.e., 0, 5, 10, and 15 wt% by the total binder, were designed. In the specimen names shown in Table 2, the terms “A1”, “A2”, and “A3” refer to the basic activator set, while the subsequent terms “CA” and “MH” refer to the additional binders, i.e., CFBC ash and Mg(OH)_2_. The trailing numbers “05”, “10”, and “15” indicate the weight ratio of the additional binders to the total binder.

### 2.2. Experimental Details

Each of the powders and water were mixed for 3 min using a 30-L capacity mixer. Note that the mortar flow measured by the ASTM C1437–15 standard for the OPC, A1, A2 and A3 mixtures, were within the ranges of 24 ± 1 cm, 18 ± 1 cm, 25 ± 1 cm and 18 ± 1 cm, respectively. The mortar specimens after casting in the molds were sealed by using plastic wrap to prevent evaporation, then cured in a chamber at the constant temperature of 20 °C.

**Figure 1:**
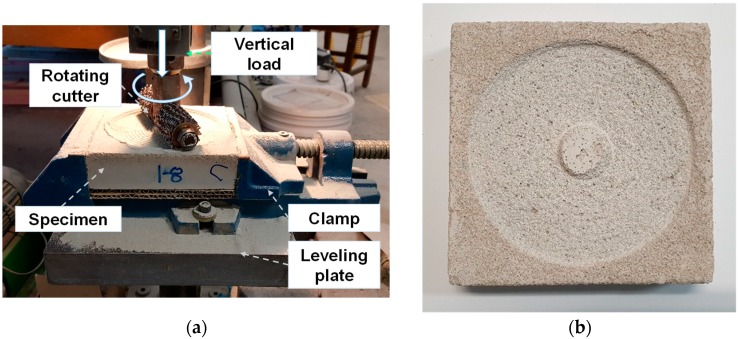
Specimen setting for abrasion resistance test (**a**) and abraded surface of specimen (**b**).

Specimens having 50 mm × 50 mm × 50 mm and 150 mm × 150 mm × 50 mm in size were prepared to measure compressive strength and abrasion resistance. The average compressive strength and abrasion resistance were calculated from three replicates. The specimens for carbonation exposure were unsealed after 28 days of curing and placed for 7 days in a carbonation chamber with the maintained condition at a temperature of 20 ± 2 °C, relative humidity of 65% and CO_2_ concentration of 5%. On the other hand, two types of non-carbonated specimens were prepared. First, to investigate the strength development over time, the specimens which were sealed cured for 3, 7, 28, and 56 days were tested. For comparison with the carbonated specimens, specimens which had been seal cured for 28 days were placed in the temperature-humidity chamber for a further 7 days. The temperature and relative humidity in the chamber was 20 ± 2 °C and 60 ± 10%, respectively, temperatures which were similar to the carbonation chamber. Hence, the drying condition was identical to that of the carbonated specimens. The compressive strength of the specimens was measured according to ASTM C109 standard. Mass losses caused by abrasion were tested according to ASTM C944. Detailed experimental setups for equipment and specimen can be found in elsewhere [18]. Figure 1 shows the specimen setting for the abrasion resistance test and the example of the abraded surface of the specimen. The vertical load applied to the specimen during the abrasion test was kept to 98 N. The depth of carbonation was measured by spraying a phenolphthalein aqueous solution. Specimens after the abrasion resistance test were cut, and cross-section of the opposite to the abraded surface was used for measuring the carbonation depth. 

## 3. Experimental Results

**Figure 2:**
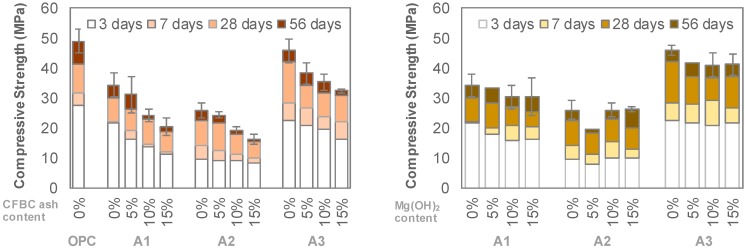
Compressive strengths of non-carbonated mixtures.

Figure 2 shows the compressive strength of non-carbonated specimens. Compared to the OPC mixture with an identical w/b, the A1 and A2 series showed lower strength while A3 series had a similar behavior on compressive strength development. However, the addition of the CFBC ash decreased the overall strength. Up to 15%, CFBC ash used in the A1 to A3 series decreased the strength to approximately 30%. On the other hand, the strength decreased slightly as the amount of Mg(OH)_2_ increased. The decrease in strength was around 10% to 15% of Mg(OH)_2_ for all AAS mixtures.

**Figure 3:**
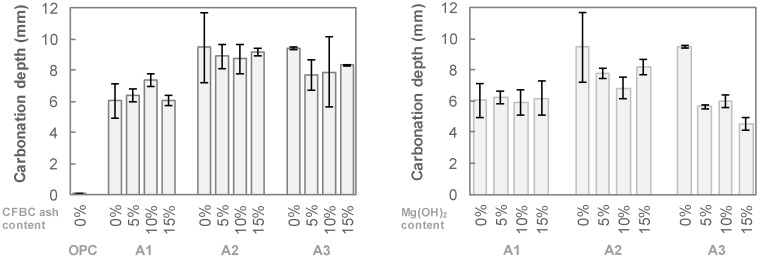
Carbonation depths of mixtures.

Figure 3 shows the depth of carbonation of the specimen after the accelerated carbonation test for 7 days. It should be noted that the measurement of the carbonation depth by spraying a phenolphthalein aqueous solution shows relatively a large deviation in some cases [19]. Even considering this, the carbonation depths of the AAS specimens ranged from 5–10 mm, while that of the OPC specimen showed less than 0.1 mm. In addition, it is observed that the carbonation depth of the A1 series was relatively thinner than those of the A2 and A3 series. As the use of CFBC ash increased to 15%, no significant change occurred to the carbonation depth for the A1 and A2 series. Whereas, when the amount of Mg(OH)_2_ increased to 15%, the carbonation depth reduced by more than 30% in the A3 series, but there was no significant change in the A1 series. The phenolphthalein aqueous solution was also sprayed on the surface of the non-carbonated AAS specimens and the color was pink. This meant that the carbonation due to carbon dioxide in the air can be neglected.

**Figure 4:**
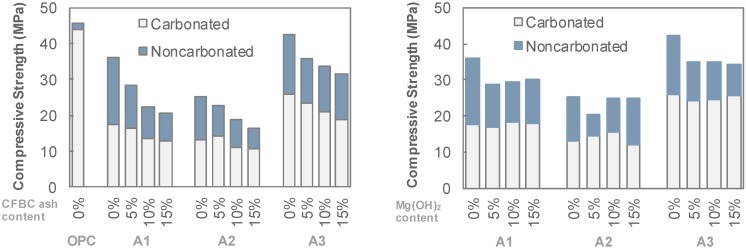
Effect of carbonation on compressive strength of specimens.

Figure 4 shows a comparison of the compressive strengths of non-carbonated and carbonated specimens. It should be noted that 50-mm cubic specimens were used for this test, and the carbonation depth of the AAS mixtures ranged from 5 to 10 mm. As a result, the strength values may be influenced by the carbonation reaction. While the strength of the OPC mixtures was not changed by carbonation, the strength of the AAS decreased in the range of 30–60%. Both the CFBC ash and Mg(OH)_2_ contents did not cause an obvious change in the strength reduction by carbonation.

**Figure 5:**
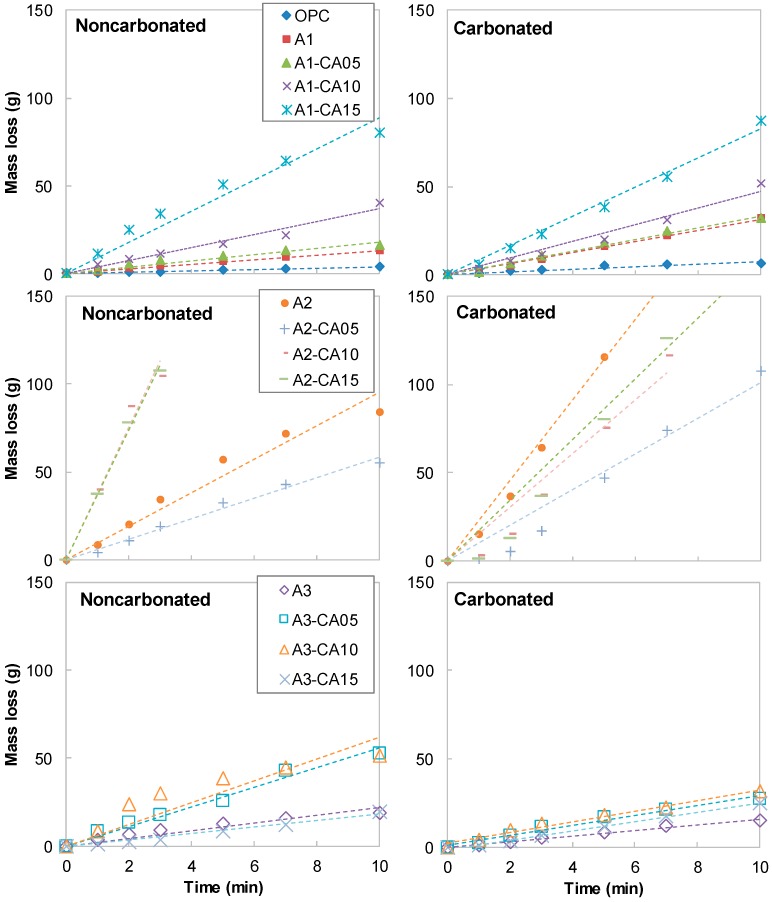

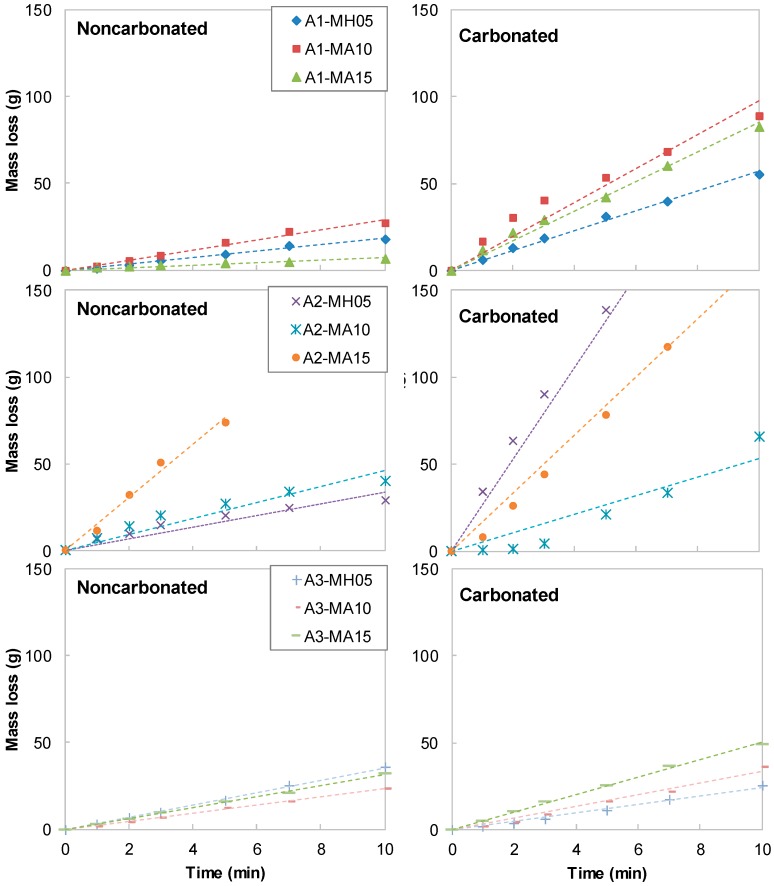
Mass loss by abrasion versus elapsed time for test.

**Figure 6:**
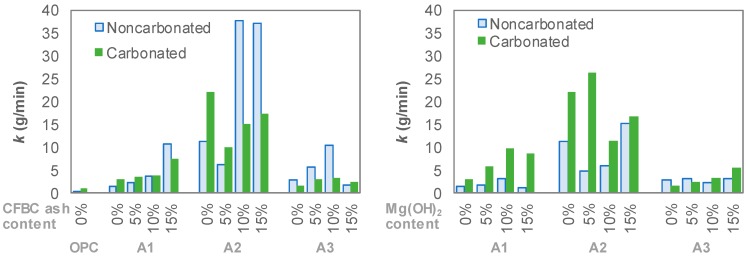
Values of parameter *k* for all mixtures.

Figure 5 shows the mass loss of the specimen over time by the abrasion test. The dotted lines in Figure 5 are the result of linear regression analysis. Considering the minor error of the abrasion test, it could be said that the relationship between mass loss and abrasion time in Figure 5 was linear in general for the most of specimens. It is well known that the relationship is given by [18]:

Abrasion mass loss (g) = *k* (g/min) × Elapsed time for abrasion (min) (1)

where *k* is a constant for each mixture. Figure 6 summarizes the *k* values obtained from the results in Figure 5. The coefficient of determination r^2^ for the linear regression analysis was greater than 0.97 in all results of the experiment. A large *k* value means that the abrasion mass loss per time was large, i.e., the abrasion resistance of the mixture was low. The *k* values of all AAS mixtures in the present study were higher than that of OPC. Although the *k* value of OPC increased more than three times from 0.39 g/min to 0.98 g/min by carbonation, these values were significantly lower compared with those of AAS. Moreover, it was confirmed that the A2 series had a significantly higher *k* value than the other series. In most mixtures, including the OPC, A1 and A2 series, the *k* values increased in carbonated specimens. In general, *k* value also increased as the amount of the CFBC ash and Mg(OH)_2_ increased. Meanwhile, in the case of the A3 series, when the CFBC ash and Mg(OH)_2_ contents were 10% or less, the *k* values decreased in carbonated specimens.

## 4. Discussion

**Figure 7:**
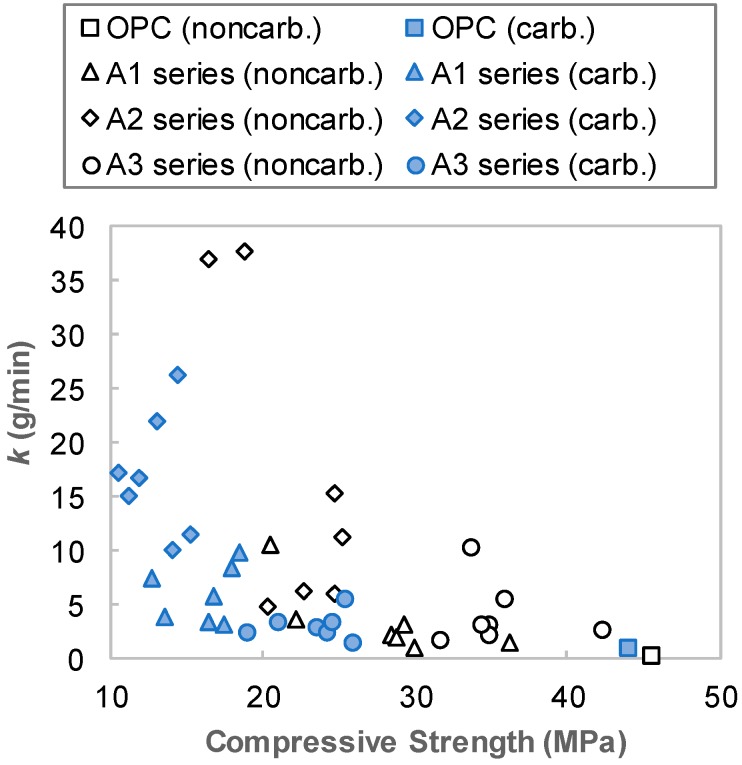
*k* value versus compressive strength of mixtures.

The experimental results can be summarized as follows. Above all, the abrasion resistance of AAS could be reduced significantly after carbonation. It is well-known that the AAS has a lower resistance to carbonation compared with OPC and its matrix strength may have decreased after carbonation [20], which is also shown in the present work. As well-known, the decrease in the strengths of the AAS matrix affected the reduction of their abrasion resistance. Although the accelerated carbonation conditions used in this study are different to the natural (ambient) carbonation condition, which produces different types of reaction products, it is clear that the performance degradation of AAS matrix due to carbonation was clearer than that of the OPC matrix [9,10,21]. Figure 7 shows plots of the compressive strength versus the *k* values of the carbonated and non-carbonated AAS specimens. Although slight differences are shown depending on the activator type, the carbonation generally resulted in both strength decreases and *k* value increases.

In the study on microstructures of AAS after carbonation conducted by Puertas et al. [19], it was found that the carbonation took place directly on the C-S-A-H gels, causing decalcification, regardless of type of activator. For AAS with sodium silicate, the decalcification of the gel prompted by carbonation led to a loss of cohesion in the AAS matrix and an increase in porosity and decline in mechanical strength [19]. This phenomenon is correlated with the results for the A2 mixture of the present work. On the other hand, for the mixture with sole sodium hydroxide, although the overall strength was about half of that with sodium silicate, the carbonation enhanced matrix cohesion, possibly as a result of the precipitation of greater amounts of calcium carbonates in the pores. It caused a reduction in total porosity and pore size and consequently an increase in mechanical strength [19]. In the present study, sodium hydroxide was combined with sodium carbonate to increase the strength (A1 mixture) similar to that of sodium silicate based AAS, but the strength decreased due to carbonation. Li et al. [22] also reported on microstructural changes and its effect on compressive strength of AAS matrices induced by carbonation. Similar to the report of Puertas et al. [19], it was observed that the strength of AAS was significantly decreased by carbonation, while that of OPC was increased slightly. In the OPC matrix, the carbonation took placed in portlandite prior to the C-S-H gel. However, the AAS having lower Ca/Si ratio compared to the OPC seldom produced portlandite and thus most of the carbonation occurred in the C-S-H gel and decreased mechanical strength [22]. A similar phenomenon was observed in a previous study conducted by the present authors of which identical materials and mixtures were used [19]. This phenomenon could have a great influence on abrasion resistance.

The abrasion resistances of AAS before and after carbonation was influenced by activator type. The AAS mixtures containing both calcium- and sodium-based activators (A1 and A3 series) had a higher abrasion resistance (*k* values of 1–10 g/min) than that with the sole sodium-based activator (A2 series, *k* values >10 g/min). Additionally, the *k* value of the A1 mixture increased from 1.47 g/min to 3.10 g/min after carbonation, while that of the A3 decreased from 2.76 g/min to 1.6 g/min. These phenomena may also be attributed to the newly formed crystals by carbonation reaction. The abrasion resistance of the A3 series was highest among the AAS series in the present work, which was approximately 1.6 times lower than that of the OPC with the same w/b. Considering that the OPC has a lower resistance against acid or sulfate, some of the AAS mixtures may have a similar or higher resistance against a complex deterioration of acid, sulfate attack, carbonation, and abrasion. Although additional binders did not alter the carbonation depth, they reduced the strength of the matrix itself and reduced the abrasion resistance.

Additionally, all of the specimens tested in the present study were cured at room temperature. The abrasion resistance may change depending on the temperature and humidity conditions of the curing, which should be evaluated in future studies.

## 5. Conclusions

The effect of carbonation on the abrasion resistance of AAS with various activators was evaluated. From a series of experiments, the following conclusions can be drawn.

(1) The resistance of AAS to carbonation and abrasion were varied sensitively with regard to the activator type. Employing a combination of Na- and Ca-based activators showed a relatively higher abrasion resistance than that of a sole Na-based activator. For the mixtures with a combination of Ca(OH)_2_, Na_2_SO_4_ and Na_2_SiF_6_ (A3 series), although the compressive strength decreased after carbonation, the abrasion resistance rather increased. Abrasion mass loss of the A3 series specimen was reduced by about 40% while the compressive strength decreased in the range of 30%.
(2) Mg(OH)_2_ and CFBC ash, i.e., additional binders tested in the present work, gave negative effects on both compressive strength and abrasion resistance of AAS mixtures. Use of these additional binders by up to 15 wt% of total binder led to a decrease in the compressive strength by approximately 30% and an increase in abrasion mass loss up to 30%.
(3) Nevertheless, the highest abrasion resistance of AAS mixtures in the present study was approximately half of that of OPC with the same w/b. The other mixtures had a very-low abrasion resistance of approximately 1/5–1/100, compared with OPC. Therefore, it can be concluded that additional caution is required when using AAS where abrasion may have occurred.

## Figures and Tables

**Table 1:** X-ray fluorescence (XRF) results of raw materials used in this study.

wt%	CaO	SiO_2_	Al_2_O_3_	Fe_2_O_3_	MgO	SO_3_	Na_2_O	Others	LOI ^a^
**Slag**	45.7	29.1	11.7	0.5	3.1	2.0	0.4	5.7	1.8
**CFBC ash**	56.5	6.2	4.6	8.5	1.8	16.9	0.1	1.8	3.6
**OPC**	60.6	23.0	3.4	3.1	3.7	3.0	-	-	2.2

^a^ Loss on ignition.

**Table 2:** Mix proportion of samples tested in this study (wt% by total binder).

Series	Slag	Basic Activator Set				Additional Binders	
		NaOH		Na_2_CO_3_		CFBC	Mg(OH)_2_
A1	93	4		3		-	-
A1-CA05	88.4	3.8		2.85		5	-
A1-CA10	83.7	3.6		2.7		10	-
A1-CA15	79.1	3.4		2.55		15	-
A1-MH05	88.4	3.8		2.85		-	5
A1-MH10	83.7	3.6		2.7		-	10
A1-MH15	79.1	3.4		2.55		-	15
**Series**	**Slag**	**Na_2_SiO_3_**				**CFBC**	**Mg(OH)_2_**
A2	90	10				-	-
A2-CA05	85.5	9.5				5	-
A2-CA10	81	9				10	-
A2-CA15	76.5	8.5				15	-
A2-MH05	85.5	9.5				-	5
A2-MH10	81	9				-	10
A2-MH15	76.5	8.5				-	15
**Series**	**Slag**	**Ca(OH)_2_**	**Na_2_SO_4_**		**Na_2_SiF_6_**	**CFBC**	**Mg(OH)_2_**
A3	88	7	3		2	-	-
A3-CA05	83.6	6.65	2.85		1.9	5	-
A3-CA10	79.2	6.3	2.7		1.8	10	-
A3-CA15	74.8	5.95	2.55		1.7	15	-
A3-MH05	83.6	6.65	2.85		1.9	-	5
A3-MH10	79.2	6.3	2.7		1.8	-	10
A3-MH15	74.8	5.95	2.55		1.7	-	15
